# COVID-19 Vaccination in Patients with Hematological Malignances

**DOI:** 10.3390/vaccines13050465

**Published:** 2025-04-25

**Authors:** Massimo Franchini, Fabrizio Maggi, Daniele Focosi

**Affiliations:** 1 Department of Transfusion Medicine and Hematology, Carlo Poma Hospital, 46100 Mantua, Italy; 2 National Institute for Infectious Diseases “Lazzaro Spallanzani”-IRCCS, 00149 Rome, Italy; fabrizio.maggi63@gmail.com; 3 North-Western Tuscany Blood Bank, Pisa University Hospital, 56124 Pisa, Italy; daniele.focosi@gmail.com

**Keywords:** hematological malignancy, SARS-CoV-2, COVID-19, vaccination, mortality

## Abstract

Patients with hematologic malignancies (HM) represent a population particularly vulnerable to infections due to their cancer-related immune deficiency and the immunosuppressive treatment they are administered. Accordingly, a high hospitalization and mortality rate has been consistently reported in such a frail population during the first COVID-19 pandemic waves. After a brief description of the clinical impact of SARS-CoV-2 infection in patients with blood cancers, this narrative review is focused on the protective effect of COVID-19 vaccines in patients with HM. All in all, the results from the literature analysis indicate that booster shots in fully vaccinated HM patients are significantly able to increase seroconversion rates, which represent the best surrogate of vaccine efficacy. Despite these encouraging data, concerns still remain regarding the lower immune responses to COVID-19 vaccines, even to booster doses, in severely immunosuppressed HM patients, such as those receiving anti-CD20 monoclonal antibody therapies and hematopoietic stem cell transplants.

## 1. Introduction

During the last five years, the coronavirus disease 2019 (COVID-19) pandemic has caused more than 770 million cases and 7 million deaths across the globe [1]. Treatment of patients hospitalized for severe acute respiratory syndrome coronavirus 2 (SARS-CoV-2) infection initially included oxygen supplementation and the use of repurposed drugs with anti-inflammatory (corticosteroids and tocilizumab), anti-thrombotic (low-molecular-weight-heparins), and antiviral (remdesivir and lopinavir/ritonavir) activity [2]. In parallel with these therapeutic agents, which represented the standard of care during the first year of the pandemic, plasma collected from recovered individuals, manufactured at transfusion centers, and containing high-titer anti-SARS-CoV-2 antibodies (COVID-19 convalescent plasma, CCP) was successfully and safely transfused to ambulatory and hospitalized COVID-19 patients during the early phase (first seven days) of the infection [3,4,5,6,7]. Apart from the subsequent introduction by pharmaceutical industries of anti-SARS-CoV-2 products, including small molecule antiviral compounds (i.e., nirmatrelvir and ritonavir, molnupiravir, etc.) and biologic immunotherapies (i.e., monoclonal antibodies and hyperimmune immunoglobulins), a huge technological advancement was represented by the marketing since the early 2021 of vaccines against SARS-CoV-2 [8,9]. While COVID-19 vaccines, which shifted the management of the pandemic from treatment towards a prophylactic approach, have shown a high efficacy in preventing or mitigating severe forms of SARS-CoV-2 infection in immunocompetent individuals [10], their benefit in immunocompromised patients, including those with hematological malignancies (HM), has been debated and remains the object of intense research. Due to their impaired immune system, immunocompromised subjects, who represent approximately 3–6% of the general population [11], are at increased risk for suboptimal responses to vaccination and thus are exposed to more severe consequences of SARS-CoV-2 infection, even with the less virulent Omicron variant [12,13,14]. In this narrative review, we explore the current literature on the effectiveness of COVID-19 vaccines in HM patients.

## 2. Search Methods

Although this narrative review is based on the authors’ expert opinions, a systematic literature search was performed through the PubMed database between December 2019 and March 2025 using the following Medical Subject Heading (MeSH) terms: “leukemia”, “lymphoma”, “myeloma”, “stem cell transplantation”, “hematological malignancy”. “hematological cancer”, “oncohematological disorder”, “immunosuppressive”, “vaccine”, “SARS-CoV-2”, and “COVID-19”.

## 3. COVID-19 in HM Patients

Patients with HM may have immune deficiencies from patient-related (i.e., age), disease-related, and treatment-related factors [15,16,17]. The latter include chemo-immunotherapies (especially B cell depleting agents), hematopoietic stem cell transplants (HSCT), and chimeric antigen receptor T-cell (CAR-T) therapy. In particular, patients with B-cell malignancies and those treated with anti-CD20 antibody-based B-cell-depleting strategies (such as rituximab, able to produce a rapid and prolonged decrease in antibody production due to complete depletion of B cells, including the memory B-cell compartment) have a profound impairment in the humoral and cellular immune response against SARS-CoV-2 [18,19,20,21]. In addition, current evidence from the literature documents that persistent viral replication in the absence of an effective immune response in immunocompromised patients, including HM patients, can lead to very high viral loads and is likely to contribute to the emergence of new SARS-CoV-2 variants of concern (VOC) [22,23,24,25,26,27]. Such immune escape may be responsible for SARS-CoV-2 resistance to anti-spike monoclonal antibodies and small molecule drugs [28,29]. Given their inability to control SARS-CoV-2 infection, resulting in prolonged viral shedding, frail immunosuppressed patients are prone to more severe COVID-19 and at an increased infection-related mortality risk [30,31,32]. Several trials and registries have investigated the clinical outcomes in HM patients with concomitant COVID-19 (the main results of the most important studies are summarized in Table 1) [33,34,35,36,37,38,39,40,41,42,43,44,45,46,47,48,49,50,51]. Interestingly, early studies showed that, among cancer patients, those with HM had the highest mortality rate [33,34]. The US COVID-19 and Cancer Consortium (CCC-19) registry showed that HM individuals were at increased risk of severe COVID-19 (adjusted odds ratio [aOR] 1.77, 95% CI 1.23–2.56) [39]. Likewise, the UK Coronavirus Cancer Monitoring Project (UKCCMP) observed that COVID-19 severity was higher in patients with HM (leukemia, lymphoma, and myeloma) compared to those with solid tumors (OR 1.57, 95% CI 1.15–2.15), but among HM, only leukemia was significantly associated with higher mortality (OR 2.25, 95% CI 1.13–4.57) [40]. The registry from the American Society of Hematology (ASH) observed an overall mortality rate of 28% in HM patients and of 42% in those hospitalized (data collected during the first pandemic wave) [41].

However, the first HM-focused studies came from Italy and Spain [37,38]. A retrospective survey conducted during the first pandemic wave in 536 HM Italian patients found an overall mortality of 37%, nearly double the one in the general COVID-19 population, and 41-fold higher than in HM patients without COVID-19 [37]: age, type of malignancy, disease status, and COVID-19 severity were independent predictors. [37]. In a population-based registry conducted in Spain and published in 2020, the mortality was 33%, being associated with higher age (>60 years), more comorbidities (>2), type of hematological malignancy (acute myeloid leukemia), and type of antineoplastic therapy (monoclonal antibodies) [38]. Again, a much lower mortality rate (10%) was found in the general Spanish population with COVID-19 [38]. Regarding the treatment of blood cancer, a retrospective study from the USA conducted during the early part of the pandemic in 77 HSCT recipients recorded hospitalization and survival rates (at 30 days) of 44% and 78%, respectively [37]. Again, 22% of all cases and 50% of those hospitalized developed severe COVID-19, with 41% of admitted individuals dying in hospital. Comorbidities and active malignancy were the main predictors of COVID-19 severity [36]. In an international analysis published in 2021 of 3801 HM patients who developed COVID-19, high hospitalization (74%) and mortality (31%) rates were observed, which peaked at 40% in patients with acute myeloid leukemia or myelodysplastic syndrome (MDS) [42]. In a Danish population-based cohort study published in 2023 on over 7000 HM patients with COVID-19, the case fatality risk (CFR) was highest during the first wave of the pandemic (3.1%) and gradually declined during Omicron [48]. Notably, the CFR was higher in patients with acute leukemia compared with those with lymphoma (6.2% versus 3%). A marked difference in COVID-19-associated mortality rate between pre-Omicron and Omicron periods (21% versus 2.3%) was also observed in a retrospective study published in the same year and conducted in Italy on 328 HM patients [50]. In a French study conducted in patients with lymphoma and published in 2021, the recent administration of anti-CD20 therapy, along with an age older than 70 years and a relapsed or refractory malignancy, was associated with a prolonged hospitalization and higher mortality risk [43]. High hospitalization and mortality rates were reported in a retrospective study from Finland in 40 patients with lymphoma receiving anti-CD20 monoclonal antibody therapy [51]. The role of recent anticancer therapy (within 12 months) in worsening COVID-19 severity was also highlighted in another study conducted in the USA in 8759 patients with B-cell lymphoid malignancy [46].

In summary, the results of the trials reported in Table 1 indicate that the clinical outcome in patients with HM and COVID-19 is influenced by various factors, including the type of neoplastic disorder (acute leukemia is associated with the worst prognosis), the pandemic period (the Omicron era is associated with a reduced hospitalization and mortality risk compared with the first pandemic waves), and the COVID-19 vaccination cycle (see next chapter). The increased risk of worse clinical outcomes of COVID-19 (measured as rates of hospitalization, intensive-care unit [ICU] admission, and mortality) in HM patients compared to the general population and to patients with other immunodeficiencies was highlighted in a systematic review analyzing 67 studies including over 10,000 HM patients [52]. Similarly, another systematic review and meta-analysis, evaluating 1186 HSCT patients from 16 studies, found a higher rate of COVID-19-related symptoms and deaths in HM patients (pooled prevalence of 79.4% and 17%, respectively) than in the general population [53].

## 4. SARS-CoV-2 Vaccination in HM Patients

Several vaccines have been developed rapidly after the emergence of the COVID-19 pandemic, including the mRNA vaccines BNT162b2 (Pfizer-BioNTech, New York, USA) and mRNA-1273 (Moderna, Cambridge, MA, USA), the non-replicating viral vector vaccines Ad26.COV2.S (Johnson & Johnson, New Brunswick, NJ, USA) and AZD1222 (Oxford-AstraZeneca, Cambridge, UK), and the inactivated vaccines Coronavac (Sinovac, Beijing, China) and BBIBP-CorV (Sinopharm Group, Shanghai, China) [54,55]. After more than 14 billion doses of COVID-19 vaccine administered worldwide, safety and efficacy are out of discussion [56]. While COVID-19 vaccination is also recommended in immunocompromised patients, several studies, especially those conducted during the first vaccination campaigns, have reported that patients with cancers, particularly while on active anticancer treatment, have an unsatisfactory response to COVID-19 vaccines, documented as an impaired seroconversion [56,57]. This finding has been more evident for HM patients, the most fragile among cancer patients, whose immune response to COVID-19 vaccines has been the object of intense research during the last four years (from December 2020 up to today) [58,59]. The majority of studies showed less efficacy in patients with HM treated with immunosuppressive drugs [60,61,62,63,64,65,66,67,68,69,70,71,72,73,74,75,76,77,78,79,80]. Table 2 summarizes the main results of the largest trials evaluating the efficacy of COVID-19 vaccines in HM patients [66,67,68,69,70,71,72,73,74,75]. The web-based registry EPICOVIDEHA (Epidemiology of COVID-19 infection in patients with hematologic malignancies: European Haematology Association) investigated SARS-CoV-2 breakthrough infections during the period January 2021 to March 2022 among 1548 vaccinated patients with HM [66]. Most patients who developed COVID-19 had lymphoid malignancies (76%) and had required a treatment for malignancy within 3 months before vaccination (68%). Ninety-one percent of the patients received 1 or 2 vaccine doses before COVID-19, and most of them were mRNA-based vaccines (89%). Overall, 60% of patients had a severe or critical infection, and hospitalization was required in 66% of patients (21% were admitted to the ICU). The overall mortality rate at 30 days after COVID-19 diagnosis was 9%, much lower than that recorded in the pre-vaccine era (31%). Multivariate analysis showed higher mortality was related to older age, active disease, critical COVID-19, and comorbidities, whereas administration of therapeutic anti-spike monoclonal antibody administration, alone or in combination with antivirals, was protective [66]. In a retrospective cohort study conducted in the USA during the period December 2020 and October 2021 on 5956 HM patients and over 500,000 controls, all fully vaccinated (two doses of mRNA vaccines or a single dose of viral vector vaccine Ad26.COV2.S) against SARS-CoV-2, the cumulative risk of breakthrough infections in HM was significantly higher than that reported in patients without malignancies (13.4% versus 4.5%, *p* < 0.001) [68]. The overall hospitalization risk for HM patients with breakthrough infections was as high as 37.8%, but only 2.2% for those without HM (hazard ratio [HR]: 34.49, 95% CI: 25.93–45.87). Similarly, the overall mortality was 5.7% for HM patients who had breakthrough infections versus 0.8% in those without [67]. In a case-control study assessing the effectiveness of the BNT162b2 mRNA COVID-19 vaccine, vaccinated HM patients had a relative risk [RR] of 1.60 for breakthrough infections (95% CI 1.12–2.37), COVID-19–related hospitalizations (RR 3.13, 95% CI 1.68–7.08), severe COVID-19 (RR 2.27, 95% CI 1.18–5.19), and COVID-19–related death (RR 1.66, 95% CI 0.72–4.47) [68]. Notably, a number of trials consistently reported the negative influence on humoral and cellular responses to COVID-19 vaccines of anti-CD20 and immunosuppressive therapies [61,62,63,64,65]. A systematic review and meta-analysis of immune response after first and second vaccine doses in adult HM patients, recruiting over 13,000 patients from 82 studies, reported a low seroconversion rate (30% and 62% after first and second vaccine doses, respectively). Anti-CD20 monoclonal antibodies and Bruton tyrosine kinase inhibitor (BTKi) therapies were associated with the lowest seroconversion rates [81]. Poor responses following the first two doses of COVID-19 vaccine were also observed in another systematic review including over 7000 HM patients from 57 eligible studies [82]. Finally, a meta-analysis including 26 studies found that although the seroconversion rates after the second vaccine dose increased compared to the first dose, they were still significantly lower than those of controls [83].

When comparing different HM subtypes, patients with myeloid malignancies tended to have better serological responses to COVID-19 vaccines than those with lymphoid cancers [11]. In a meta-analysis of 64 studies comprising 8546 HM patients, serological responses were 83% for acute leukemia, 81% for myeloproliferative neoplasms (MPN), and 63% for MDS, higher than those observed in patients with chronic lymphocytic leukemia (CLL) and other lymphoid malignancies (44% and 52%, respectively) [84]. Among patients with lymphoid cancers, those treated with B-cell-depleting therapies had the lowest response to the COVID-19 vaccine. Similar results were shown in another meta-analysis [85].

Due to the unsatisfactory response to the full vaccination cycle, several trials investigated seroconversion rates in HM patients following booster doses during the pandemic period. The first prospective study of a booster dose in HM patients demonstrated a seroconversion rate of 56%, even in patients who recently interrupted chemotherapy [71], although anti-CD20 treatment was significantly associated with inferior post-booster seroconversion, and all of the persistently seronegative patients had B-cell malignancies [70]. Another study observed that a significant proportion (65%) of HM patients who did not make detectable antibodies after initial vaccination were able to produce antibodies after booster vaccination [71]. In a cohort of 584 HM patients, the booster dose of COVID-19 vaccine significantly improved the seroconversion rate (78.8%) and antibody titers, which were comparable to healthy individuals [72]. Notably, the neutralizing capacity was improved also against the most recent VOC, including Omicron. Patients receiving or shortly after completing anti-CD20 or BTKi therapies and HSCT were less or not responsive to the third vaccine dose and thus needed to be revaccinated after treatment or transplantation [72]. Interestingly, an Italian study showed that the booster vaccine dose was able to improve both humoral and T-cell immune response in HM patients [73]. Another study, including 536 immunocompromised individuals, found that seropositivity following the third COVID-19 mRNA vaccine dose was lowest for recipients of solid organ transplants and HM patients [75]. A case-control study evaluating a large population (over 6000) of vaccinated HM patients observed that the risk of severe COVID-19 remained high also during the Omicron variant wave [76]. The booster vaccine dose and the concomitant antineoplastic or immunosuppressive treatment appeared to decrease and increase such risk, respectively [76]. Finally, a prospective observational study conducted in France on 43 patients with lymphoid malignancies showed that the third vaccine dose was able to increase neutralizing antibody titers in seropositive patients, but not in seronegative patients, after the first two vaccine doses [69]. A systematic review and individual patient data meta-analysis of 22 studies, pooling a total of 849 HM patients and 82 patients with solid cancers, showed that 44% of HM patients whose serological response remained negative after full COVID-19 vaccination could experience seroconversion after the booster shot. This seroconversion rate was, however, approximately half of that reached by patients with solid cancers (80%) [73]. A recent systematic review on allogeneic HSCT recipients, which included 385 patients from 7 studies, reported a pooled humoral response rate of 74% following three doses of COVID-19 vaccine [86]. Furthermore, in approximately half of the patients who did not seroconvert with the two-dose full vaccination cycle, an immune response against the virus was observed [86]. In any case, the antibody response after the third booster vaccination in patients with hematological neoplasms was lower than that obtained in those with solid cancers, as documented by another systematic review [87,88].

More recently, a number of trials have evaluated the effect of additional booster doses (4th and 5th doses) in HM patients. In a study conducted in Italy on 71 HM patients, it was found that the fourth COVID-19 vaccine increased the levels of circulating anti-receptor binding domain (RBD) antibodies in all the patients, with the exception of those undergoing B-cell targeted therapies. Notably, the antibody response elicited by the vaccine was predominantly directed against the wild type (WT) and to a much lower extent to the Omicron VOC BA.2 [74]. In a study on 372 patients with HM, of whom 90% received a 4th vaccine dose and 40% received a 5th vaccine dose, serologic responses were 77% and 91%, respectively. One-third of the cohort contracted COVID-19 infection: only four patients were hospitalized, and no COVID-19-related deaths were reported [77]. Interestingly, the neutralizing antibody responses to SARS-CoV-2 VOC after additional booster doses increased also in HSCT patients [55], as shown by a cohort study evaluating 77 allogeneic HSCT recipients, where anti-RBD antibody titers increased further (from 13,300 units/mL to 34,700 units/mL) among those patients who received a fourth vaccine dose [78]. In a study on 60 patients with hematologic malignancy who received CAR-T therapy, the seroconversion rates were significantly higher after four or more vaccine doses (OR 8.2, *p* = 0.008) [79].

Based on the literature results, several oncohematological scientific societies provided COVID-19 vaccine recommendations for HM patients, which have been continuously updated according to newer viral VOCs and vaccine products [89,90]. The 2022 European Conference of Infections in Leukemia (ECIL 9) guidelines recommend HM patients to receive 3 doses of mRNA vaccines or 2 doses of a protein subunit vaccine [89]. The recommended timing of vaccination is to vaccinate before starting active treatment, or during maintenance therapy or the off-therapy phase, or before HSCT. Patients who have been vaccinated before or during hematological treatment should be tested for antibody titers at three to six months after the end of treatment, and, if the titers are low, they should be revaccinated [89]. Additional vaccine doses should be considered at least 3 or at least 4 months after COVID-19 infection.

## 5. Conclusions

There is consistent evidence from the literature that HM patients are at high risk of contracting COVID-19 and suffering its deleterious consequences, whose management represents a serious challenge for hematologists. Indeed, the immune dysfunction typical of this category of fragile patients makes them particularly susceptible to COVID-19-related severe illness and mortality. Although the advent of the Omicron variant has significantly mitigated the virulence of SARS-CoV-2 infection, reducing its mortality risk, a substantial proportion of HM patients continues to contract the more severe forms of COVID-19. The booster doses (>3) of COVID-19 vaccine are able to increase the suboptimal response to the full vaccination cycle in HM patients, but lower seroconversion rates have still been detected in subsets of patients, particularly those receiving anti-CD20 monoclonal antibodies, HSCT, or CAR-T therapies.

## Figures and Tables

**Table 1:** Clinical outcomes of the main studies in COVID-19 patients with hematological malignancies.

First Author [ref.], Year	Country, Study Period ^1^	Study Design	Population (n)	Hematologic Malignancy	Main Findings
Dai [34], 2020	China, from 2020-01 to 2020-02	Cohort study	105 with cancer (8 with HM)	NA	Among cancer patients, those with HM had the highest death rate (33.3%) and ICU admission rate (44.4%).
Mehta [35], 2020	USA, from 2020-03 to 2020-04	Cohort study	218 with cancer (54 with HM)	37% Ly, 10% AL, 24% MM, 10% MDS, 13% MPN, 6% CLL	Mortality was higher in HM patients than in those with solid malignancies (37% versus 25%).
Garcia-Suarez [36], 2020	Spain, from 2020-02 to 2020-05	Population-based registry	697	27% Ly, 9% AL, 20% MM, 16% CLL, 9% MPN, 11% MDS	A 33% mortality rate was observed. Age > 60 years, > 2 comorbidities, and antineoplastic treatment with monoclonal antibodies were independent prognostic factors for mortality.
Shah [37], 2020	USA, from 2002-03 to 2020-05	Retrospective study	77	31% Ly, 36% MM, 5% MDS; 45.5% allo-HSCT; 48% auto-HSCT; 6.5% CAR T	Forty-four percent of patients were hospitalized. Overall survival at 30 days was 78%. The presence of comorbidities (>2) and of an active hematologic malignancy predicted increased COVID-19 severity.
Passamonti [38], 2020	Italy, from 2020-02 to 2020-05	Retrospective study	536	44% Ly, 13% AL, 20% MM, 15% MPN, 8% MDS	A higher SMR (2.04) was recorded in HM patients compared with the Italian general population with COVID-19. Older age, progressive disease status, diagnosis of AL or aggressive Ly, and severe or critical COVID-19 were associated with worse overall survival.
Piñana [39], 2020	Spain, from 2020-03 to 2020-05	Retrospective study	367	28% Ly, 24% AL, 16% MM, 10% CLL, 11% MPN, 5% MDS	The overall mortality rate was 29%. Older age (>70 years), advanced HM, and neutropenia were independently associated with increased overall mortality.
Kuderer [40], 2020	USA, from 2020-03 to 2020-04	Cohort study (registry)	928 with cancer (204 with HM)	47% Ly, 9% AL, 27% MM	Among cancer patients, patients with HM had an increased risk of severe COVID-19.
Lee [41], 2020	UK, from 2020-03 to 2020-05	Cohort study (registry)	1044 with cancer (224 with HM)	NA	HM patients had more severe COVID-19 compared to those with solid tumors. AL was associated with a higher mortality rate.
Wood [42], 2020	USA-Canada from 2020-04 to 2020-07	Cohort study (registry)	250	31% Ly, 33% AL, 12% CLL, 16% MM; 10% MPN	Mortality rates of 28% and 42% were observed in all hospitalized COVID-19 HM patients.
Pagano [43], 2021	International, from 2020-03 to 2020-12	Web-based registry	3801	33% Ly, 18% AL, 18% MM, 4% CLL, 11% MPN, 7% MDS	The rate of hospitalization was 74%. The overall mortality rate was 31%. The mortality rate significantly decreased between the first COVID-19 wave (March–May 2020) and the second wave (October–December 2020) (40.7% versus 24.8%, *p* < 0.0001).
Dulery [44], 2021	France, from 2020-03 to 2020-04	Retrospective study	111	100% Ly	Recent anti-CD20 therapy, older age (>70 years), and relapsed/refractory Ly were associated with prolonged LOS and higher risk of death.
Ljungman [45], 2021	Europe, from 2020-02 to 2020-07	Retrospective study	382	146 auto-HSCT, 236 allo-HSCT	Older age and a moderate/high immunodeficiency index increased the mortality risk.
Sharma [46], 2021	USA, from 2020-03 to 2020-08	Observational cohort study	318	134 auto-HSCT, 184 allo-HSCT	Age older than 50 years and male sex represented mortality risk factors among allo-HSCT recipients.
Rubinstein [47], 2022	USA, from 2020-03 to 2021-06	Retrospective study	8759	100% Ly (B-lymphoid malignancy)	A 55% hospitalization rate was recorded. The mortality rate was 13%. Recent therapy (<12 months) for B-lymphoid malignancy was an independent risk factor for COVID-19 severity.
Lee [48], 2022	USA, from 2020-03 to 2021-02	Retrospective study	382	100% Ly (with persistent COVID-19 infection)	A 22% mortality rate was observed in hospitalized patients. Cardiovascular disease, active treatment, and CAR T-cell therapy were independently associated with mortality.
Lund [49], 2023	Denmark, from 2020-02 to 2023-04	Cohort study	7154	43% Ly, 12% AL, 15% MM, 12% CLL, 18% MPN	The rate of SARS-CoV-2 vaccinated patients (first dose) was 89%. A CFR of 3.1% was detected. The highest CFR (6.2%) was observed in AL patients. The rate of hospitalization was 21.7%.
Zhu [50], 2023	China, 2022-12	Retrospective study	412	13% Ly, 38% AL, 22% MM, 24.5% MPN, 1.5% MDS	Patients with advanced malignancies had a higher mortality rate compared with those with stable malignancy (10.5% versus 0%, *p* < 0.001).
Mikulska [51]. 2023	Italy, from 2021-03 to 2022-07	Retrospective study	328	38% Ly, 20% AL, 24% MM, 10% CLL, 3% MDS	COVID-19-associated mortality was 3.4% (21% in pre-Omicron and 2.3% during the Omicron period). Independent predictors of mortality were older age, AL diagnosis, hematological malignancy (AL or MDS), and the pre-omicron period.
Feuth [52], 2024	Finland, from 2020-03 to 2023-03	Retrospective study	40	100% Ly (patients on anti-CD20 monoclonal antibody therapy)	In this study, 59.5% of patients were hospitalized for COVID-19. COVID-19-related mortality was 17.5%. Anti-CD20 monoclonal antibody therapy was associated with a high hospitalization and mortality risk in Ly patients.

Abbreviations: Ly, lymphoma; AL, acute leukemia; MM, multiple myeloma; CLL, chronic lymphocytic leukemia; MPN, myeloproliferative neoplasm; MDS, myelodysplastic syndrome; CFR, case fatality risk; LOS, length of stay; allo-HSCT, allogeneic hematopoietic cell transplantation; auto-HSCT, autologous hematopoietic cell transplantation; CAR T, CD19-directed chimeric antigen receptor T cell therapy; SMR, standardized mortality ratio; HM, hematological malignancy; NA, not available; ICU, intensive care unit. ^1^ year-month.

**Table 2:** Results of the main studies evaluating the efficacy and safety of COVID-19 vaccines in patients with hematological malignancies.

First Author [ref.], Year	Country, Study Period ^1^	Study Design/Vaccine Type	Population (n)	Hematologic Malignancy	Main Conclusions
Pagano [67], 2022	International, from 2021-01 to 2022-03	Web-based registry/mRNA (89%), vector-based (9%), inactivated (2%)	1548	40% Ly, 13% AL, 18% MM, 6% MDS, 14% CLL, 9% MPN	Although COVID-19 mortality was significantly lower than in the prevaccination era, it remained considerably high. The death rate was lower in patients who received anti-spike monoclonal antibodies alone or in combination with antivirals.
Wang [68], 2022	USA, from 2020-12 to 2021-10	Retrospective cohort study/ mRNA (99%), vector-based (1%)	5956	53% Ly, 13% AL, 20% MM, 17% CLL, 5% MPN	Among the fully vaccinated population, HM patients had a significantly higher risk for breakthrough infections compared to patients without cancer, which drove hospitalizations and mortality.
Mittelman [69], 2022	USA, from 2020-12 to 2021-02	Case-control study/mRNA BNT162b2	32,516	NA	Vaccinated HM patients, in particular those receiving treatment, suffer from adverse COVID-19 outcomes more than vaccinated individuals with intact immune systems.
Re [70], 2022	France,	Prospective cohort study/mRNA BNT162b2	43	33% CLL, 32% Ly, 35% MM	The results of the study support the third vaccine dose in patients with lymphoid malignancies, although the proportion of patients still seronegative after the full vaccination cycle (42%) did not respond to the booster dose.
Shapiro [71], 2022	USA, from 2021-08 to 2021-12	Cohort study/mRNA (95%), vector-based (5%)	57	16% MyM, 61% LyM, 25% MM	An additional (booster) vaccine dose increases the response rate (56%) in HM patients. Patients with lymphoid malignancies, particularly those receiving anti-CD20 therapies, had the lowest seroconversion rate.
Haggenburg [73], 2022	Holland, 2022	Prospective observational cohort study	584	17% Ly, 27% MM, 13% CLL, 18% MPN, 15% HSCT	The primary COVID-19 vaccination schedule for immunocompromised patients with HM should be supplemented with a delayed third vaccination.
Resigno [75], 2023	Italy, from 2021-10 to 2022-06	Cohort study/ mRNA	71	NA	The fourth vaccine dose increased the levels of neutralizing anti-SARS-CoV-2 antibodies in all patients with the exception of those undergoing B-cell targeted therapies.
Haidar [76], 2024	USA, from 2021-04 to 2021-07	Retrospective cohort study/ mRNA	80	28% Ly, 1% AL, 14% MM, 36% CLL, 5% MPN, 3% MDS	HM patients had suboptimal responses to the third COVID-19 mRNA vaccine dose.
Anand [77], 2024	USA, from 2020-01 to 2023-04	Case-control study/ mRNA (95%) vector-based (5%)	6122	61% Ly, 3% AL, 8% MDS, 20% CLL, 22% MPN	HM patients remain at high risk for severe COVID-19 in the event of infection with SARS-CoV-2 after vaccination. This risk was increased by active treatment with antineoplastic or immunosuppressive drugs and was reduced by booster vaccine doses.
Bhella [78], 2024	Canada, from 2021-08 to 2022-06	Prospective observational study /mRNA	372	29% Ly, 10% CLL, 23% MM, 10% AL, 10% MPN, 25% HSCT	Humoral immune response improved with subsequent doses (3rd and 4th) of COVID-19 vaccines.

Abbreviations: NA, not evaluated; Ly, lymphoma; AL, acute leukemia; MM, multiple myeloma; CLL, chronic lymphocytic leukemia; MPN, myeloproliferative neoplasm; MDS, myelodysplastic syndrome; MyM, myeloid malignancy; LyM, lymphoid malignancy; HSCT, hematopoietic cell transplantation. ^1^ year-month.

## Data Availability

No new data were created or analyzed in this study. Data sharing is not applicable to this article.

## Abbreviations

COVID-19: coronavirus disease 2019; CCP: COVID-19 convalescent plasma; CLL: chronic lymphocytic leukemia; ICU: intensive care unit; HM: hematologic malignancy; HR: hazard ratio; MDS: myelodysplastic syndrome; SARS-CoV-2: severe acute respiratory syndrome coronavirus 2; VOC: variant of concern.
